# Pedunculated Hemangioma in the Left Ventricle: Case Report in an Asymptomatic Young Patient

**DOI:** 10.21470/1678-9741-2025-0053

**Published:** 2026-02-13

**Authors:** George Ronald Soncini da Rosa, Paulo Loffy, Danielle Giacometti Sakamoto, Diego Destro da Silva, Nathalia Recalcatti Crestani, Patrícia Hau F. dos Santos Timotheo, Helena Pierdoná, Bárbara Fadani Schmitz

**Affiliations:** 1 Cirurgia Cardiovascular, Complexo Hospitalar de Clínicas da Universidade Federal do Paraná, Curitiba, Paraná, Brazil; 2 Cirurgia Cardiovascular, Hospital Sugisawa, Curitiba, Paraná, Brazil; 3 Cirurgia Cardiovascular e Cardiologia, Hospital Sugisawa, Curitiba, Paraná, Brazil; 4 Cirurgia Cardiovascular, Universidade do Contestado, Mafra, Santa Catarina, Brazil

**Keywords:** Heart Neoplasms, Hemangioma, Incidental Findings, Doppler Echocardiography.

## Abstract

Cardiac hemangiomas are benign neoplasms of extremely rare occurrence and, in most cases, asymptomatic, commonly detected incidentally during examinations performed for other clinical indications. To confirm the diagnosis and prevent potential complications, surgical excision followed by histopathological analysis is recommended. In this case report, we describe a left ventricular hemangioma incidentally identified during an echocardiographic examination.

## INTRODUCTION

Cardiac hemangiomas are extremely rare benign tumors^[1,2]^, often identified incidentally during imaging exams, such as echocardiography, performed for other reasons^[3-5]^. Although they may be asymptomatic, these tumors have the potential to cause severe complications, including arrhythmias, dyspnea, and even sudden death^[2,6,7]^. Surgical intervention is recommended for both diagnostic confirmation and the prevention of significant complications^[1,2,8,9]^.

## CASE PRESENTATION

A 20-year-old black male patient presented for a consultation complaining of malaise, occasional resting dyspnea, and a recent increase in the frequency of palpitations. His medical history was unremarkable, he denied the use of medications and reported engaging in physical activity five times a week, focusing on strength training. Regarding family history, he mentioned that his grandmother had a history of myocardial revascularization.

Upon admission, his vital signs were as follows: blood pressure 140 x 100 mmHg, with normal cardiac auscultation. The electrocardiogram was within normal limits. The patient also underwent a stress test, which revealed no abnormalities.

### Surgical Technique

**Fig. 1:**
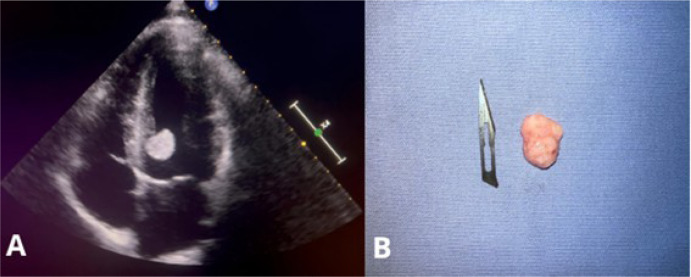
(A) Transthoracic echocardiogram revealing a mass in the left ventricle; (B) excised mass.

Transthoracic echocardiography revealed an oval-shaped, pedunculated, and mobile mass measuring 18 x 16 mm, located near the interventricular septum close to the left ventricular outflow tract (Figure 1A), with a primary suspicion of myxoma. Additionally, other structures such as valves and cardiac chambers showed no abnormalities.

**Fig. 2:**
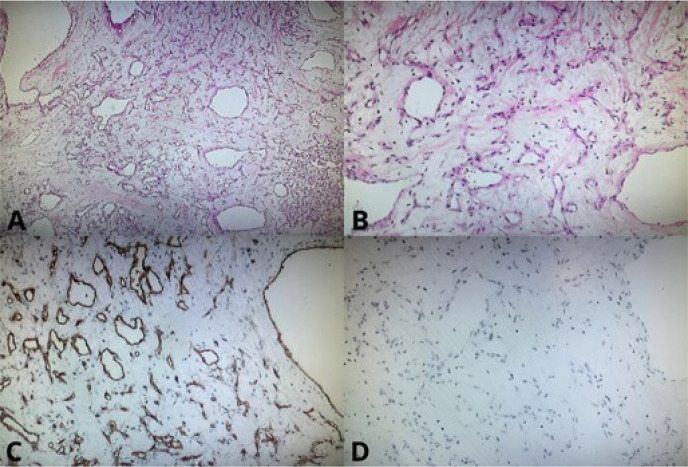
(A) Photomicrograph showing endothelial immunostaining (CD34) composed of a vascular network (CD34 x 100). (B) Photomicrograph showing no immunostaining for mucin (Mucin4), ruling out a diagnosis of myxoma (Muc4 x 100). (C) Photomicrograph showing a mesenchymal neoplasm composed of loose stroma with proliferation of vessels of varying diameters, without endothelial atypia (HE x 40). (D) Photomicrograph showing a mesenchymal neoplasm composed of loose stroma with proliferation of vessels of varying diameters, without endothelial atypia (HE x 100).

The patient subsequently underwent surgery for resection of the intracardiac tumor. The excised mass was nodular in shape, with a pinkish and dull surface, measuring 2 x 1.2 cm. On sectioning, it appeared soft and elastic (Figure 1B). Histopathological examination confirmed the diagnosis of a vascular-rich mesenchymal neoplasm. Furthermore, the sample designated for immunohistochemical study consisted of fragments of a lesion composed of numerous irregular vascular channels, some dilated and filled with red blood cells, lined by endothelial cells without atypia, in a mixed cavernous and capillary pattern. The analysis also revealed CD34 expression, corroborating the diagnosis of cardiac hemangioma (Figure 2).

The patient experienced no postoperative complications and was followed up with transthoracic echocardiography 37 days later, which revealed normal functions and dimensions.

## DISCUSSION

Cardiac hemangiomas are rare in adults, comprising approximately 2.8% of primary cardiac tumors^[1,2]^. They are benign in nature and histologically identical to those that occur elsewhere in the body^[1]^.

They are generally asymptomatic and often discovered from the fifth decade of life during the evaluation of other cardiac conditions, frequently leading to alternative diagnoses^[2]^. When symptomatic, clinical manifestations depend on the tumor's location and progression, which may include dyspnea, arrhythmias, heart failure, pericardial effusion, tamponade, and even sudden death^[2,6,7]^.

Cardiac tumors are rarely encountered, as it is uncommon for patients in this age group with healthy lifestyles and regular physical activity to seek cardiology consultations. Another key point is that, although histologically benign, their behavior may be malignant, with a potential for sudden death due to their thin, unstable pedunculated nature, posing a risk of detachment^[5,7,10]^. The movement of the hemangioma within the ventricle is the primary cause of palpitations, with a risk of entanglement in the chordae tendineae, potentially leading to acute mitral insufficiency^[4,7,9]^. While in many cases, hemangiomas are only discovered postmortem during autopsies following sudden death^[1,2,10]^, the active investigation of this patient's clinical condition was crucial for early diagnosis, enabling effective treatment and improving the prognosis.

Echocardiography is one of the most widely used examinations for evaluating and diagnosing intracardiac tumor masses due to its ability to provide real-time imaging, being noninvasive, and having low cost^[3,4]^. Additionally, magnetic resonance imaging and computed tomography can be used to estimate the tumor's extent and location^[2,6]^, while angiography may be employed to assess the involved vessels^[5,8]^.

Intracavitary tumors resemble other tumors and are indistinguishable by echocardiography, with the diagnosis of hemangioma only confirmed after mass extraction and histopathological analysis^[1,2]^. In addition to being frequently mistaken for myxomas, other differential diagnoses for hemangiomas include cardiac vegetations, some metastatic tumors, primary benign or malignant tumors, and thrombi resulting from atrial fibrillation^[2,9]^.

After resection of the intracardiac tumor and confirmation of the hemangioma diagnosis through histopathological examination, the patient was reevaluated, showing the left ventricle with normal dimensions, contractility, global systolic function, and diastolic function.

## CONCLUSION

This article highlights the rarity of left ventricular hemangiomas, particularly in young patients, and underscores the importance of prompt medical attention and detailed clinical investigation, both of which are essential for a favorable prognosis.

## Data Availability

The authors declare that data sharing is not applicable to this article as no new data were created or analyzed.

## Artificial Intelligence Usage

The authors declare that no artificial intelligence tool was used in the preparation of this article.
